# Complete mitochondrial genome of a Eurytopic midge, *Polypedilum nubifer* (Diptera: Chironomidae)

**DOI:** 10.1080/23802359.2022.2122746

**Published:** 2022-11-11

**Authors:** Yun-Li Xiao, Zi-Gang Xu, Jia-Xin Wang, Xiang-Liang Fang, Yue Fu

**Affiliations:** a Hubei Key Laboratory of Economic Forest Germplasm Improvement and Resources Comprehensive Utilization, Hubei Collaborative Innovation Center for the Characteristic Resources Exploitation of Dabie Mountains, Hubei Zhongke Research Institute of Industrial Technology, College of Biology and Agricultural Resources, Huanggang Normal University, Huanggang City, Hubei, P. R. China; b College of Agriculture, Yangtze University, Jingzhou City, Hubei, P. R. China

**Keywords:** Chironomoidea, *Polypedilum*, phylogenetic relationship

## Abstract

The complete mitochondrial genome of *Polypedilum nubifer* was sequenced and annotated, and its general features and base composition were analyzed. The mitogenome was 15,896 bp long, comprising 13 protein-coding genes, 22 transport RNA genes, 2 ribosomal RNA genes, and 1 control region. The phylogenetic relationships based on the concatenated nucleotide sequences of 17 metagenomes from families Chironomidae, Ceratopogonidae, and Simuliidae were reconstructed. According to the phylogenetic topologies, *P. nubifer* was closely related to (*P. unifascium* + *P. vanderplanki*) in the maximum likelihood tree.

*Polypedilum nubifer* (Skuse 1889), belonging to the subfamily Chironominae, is a widely distributed and recorded species in Australia, the Federated States of Micronesia, Asia, Europe, Africa, Hawaii, and Florida. In China, the species has been found in the Palearctic (Liaoning, Ningxia, Inner Mongolia, Tianjin, Henan, Shananxi, Gansu, Anhui, and Xinjiang) and Oriental (Sichuan, Yunnan, Fujian, Taiwan, Guizhou, Guangdong, Guangxi, and Hainan) regions (Wang et al. 2020). Most larvae of the genus *Polypedilum* grow in contaminated water. Thus, *P. nubifer* can be used as an indicator species for monitoring water environments (Paine and Gaufin 1956; Armitage et al. 1995). *Polypedilum nubifer* is a common nuisance midge found in the tropical and subtropical waters in the Afrotropical, Palearctic, Oriental, Australasian, and Nearctic regions. Populations can become extremely abundant in warm, shallow, and eutrophic waters subjected to seasonal drying (Tabaru et al. 1987; Trayler et al. 1994; Cranston 2007; Jacobsen and Perry 2007; Cranston et al. 2013).

The specimen in this study was collected from the Wujiashan Mountains (31°0845′N, 115°8189′E), altitude 487 m, Dahechong Village, Yingshan County, Huanggang City, Hubei Province, CHINA) on 9 August 2020 using a light trap. Samples were collected by Yue Fu (email: fuyue20190125@163.com) and deposited in the Biodiversity Herbarium of Huanggang Normal University (http://shengwu.hgnu.edu.cn/2018/1130/c435a7076/page.htm, Yue Fu) under the voucher number HGNU-Ydbs152. Whole genomic DNA was extracted using the DNeasy Animal Tissue Kit (Tiangen, China), and the mitochondrial genome was sequenced using the Illumina HiSeq X System. Reads were assembled using NOVOPlasty (Dierckxsens et al. 2016), and the mitochondrial genome was assembled using SPAdes (version v3.11.1; Bankevich et al. 2012). Phylogenetic analyses were reconstructed using PhyloSuite (Zhang et al. 2020) with several plug-in programs including MAFFT using the ‘–auto’ strategy for multiple sequence alignment (Katoh and Standley 2013). The AICc criterion in PartitionFinder2 was used to select best-fit partitioning schemes and models (Lanfear et al. 2017). The phylogenetic tree was inferred using IQ-TREE (Minh et al. 2013; Nguyen et al. 2015) and edited in iTOL (Letunic and Bork 2019).

The complete mitogenome of *P. nubifer* was 15,896 bp long (GenBank accession number: MZ747090). It consists of 13 protein-coding genes (PCGs), 22 tRNA genes, and 2 rRNA genes (totaling 37 genes), and 1 control region. The genomic nucleotide composition was 40.07% A, 37.55% T, 13.05% C, and 9.33% G. The total length of the 13 PCGs in the mitochondrial genome was 11,196 bp. Four genes had overlapping regions, with a total overlapping length of 18 bp. The two longest overlapping regions (7 bp) were located between atp8/atp6 and nad4/nad4l. There were 26 intergeneric spacers with a total length of 537 bp, ranging from 1 to 87 bp. The longest intergenic region was located between *trnA* and *trnR*. The initiation codons of PCGs complied with the ATN rule: six genes (*cox2, atp6, cox3, nad4, nad4l,* and *cob*) with an ATG start codon, two genes (*atp8* and *nad3*) with an ATA start codon, one gene (*nad2*) with an ATT start codon, one gene (*nad6*) with an ATC start codon, and two genes (*nad5* and *nad1*) with a GTG start codon. All PCGs had a TAA stop codon. The length of the tRNA genes ranged from 65 to 72 bp, with a total length of 1,487 bp. The lengths of the 12S rRNA and 16S rRNA were 810 and 1,430 bp, respectively.

Maximum likelihood analysis showed species of the same family clustered together (Figure 1). *P. nubifer* and (*P. unifascium* + *P. vanderplanki*) were clustered together with a bootstrap score of 99.8%; all of them belong to genus *Polypedilum*. Further, *P. nubifer* + (*P. unifascium* + *P. vanderplanki*) were closely related to *Chironomus flaviplumus* + *Microchironomus tabarui*; all of them belong to tribe Chironomini, subfamily Chironominae, which was in accordance with traditional morphological classification.

**Figure 1.**
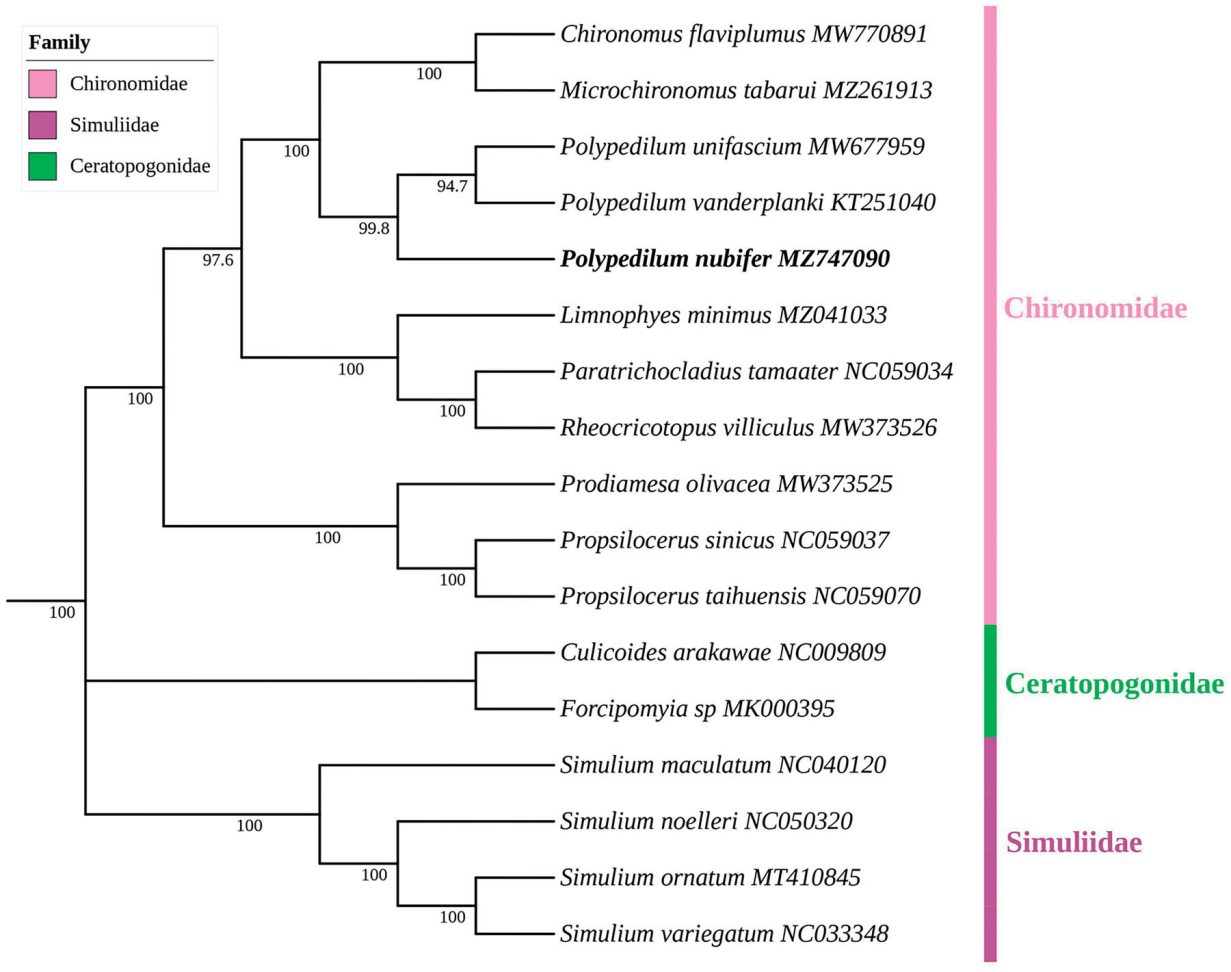
Maximum likelihood phylogenetic tree generated based on 37 genes from mitogenomes of 17 Chironomoidea species. Ceratopogonidae and Simuliidae are outgroups.

## Data Availability

The data that was obtained at this study are available in the NCBI under the accession number MZ747090 (https://www.ncbi.nlm.nih.gov/nuccore/MZ747090). The associated BioProject, Bio-Sample numbers, and SRA are PRJNA804390, SAMN25889263, and SRR18003347, respectively.
